# DNA methylation reprogramming of functional elements during mammalian embryonic development

**DOI:** 10.1038/s41421-018-0039-9

**Published:** 2018-08-07

**Authors:** Congru Li, Yong Fan, Guoqiang Li, Xiaocui Xu, Jialei Duan, Rong Li, Xiangjin Kang, Xin Ma, Xuepeng Chen, Yuwen Ke, Jie Yan, Ying Lian, Ping Liu, Yue Zhao, Hongcui Zhao, Yaoyong Chen, Yang Yu, Jiang Liu

**Affiliations:** 1 Ministry of Education Key Laboratory of Assisted Reproduction, and Beijing Key Laboratory of Reproductive Endocrinology and Assisted Reproductive Technology, Center of Reproductive Medicine, Peking University Third Hospital, 100191 Beijing, China; 2 CAS Key Laboratory of Genome Sciences and Information, Collaborative Innovation Center of Genetics and Development, Beijing Institute of Genomics, CAS, 100101 Beijing, China; 3 University of Chinese Academy of Sciences, 100029 Beijing, China; 4 Key Laboratory for Major Obstetric Diseases of Guangdong Province, Third Affiliated Hospital of Guangzhou Medical University, 510150 Guangzhou, China

## Abstract

DNA methylation plays important roles during development. However, the DNA methylation reprogramming of functional elements has not been fully investigated during mammalian embryonic development. Herein, using our modified MethylC-Seq library generation method and published post-bisulphite adapter-tagging (PBAT) method, we generated genome-wide DNA methylomes of human gametes and early embryos at single-base resolution and compared them with mouse methylomes. We showed that the dynamics of DNA methylation in functional elements are conserved between humans and mice during early embryogenesis, except for satellite repeats. We further found that oocyte-specific hypomethylated promoters usually exhibit low CpG densities. Genes with oocyte-specific hypomethylated promoters generally show oocyte-specific hypomethylated genic and intergenic regions, and these hypomethylated regions contribute to the hypomethylation pattern of mammalian oocytes. Furthermore, hypomethylated genic regions with low CG densities correlate with gene silencing in oocytes, whereas hypomethylated genic regions with high CG densities correspond to high gene expression. We further show that methylation reprogramming of enhancers during early embryogenesis is highly associated with the development of almost all human organs. Our data support the hypothesis that DNA methylation plays important roles during mammalian development.

## Introduction

Epigenetic information plays critical roles during animal development^1–4^. The plasticity of the epigenome enables cell differentiation, organogenesis and animal development. Proper epigenomic patterns are also required to ensure the totipotency of early embryos of animals^2^. One key epigenetic modification found in most plants, animals and fungal models is 5mC^5^, which has a profound impact on genome stability, gene expression and development^6–8^.

Recent studies have shown that the sperm DNA methylome can be stably inherited by early embryos in zebrafish^9,10^, indicating the existence of transgenerational epigenetic inheritance in animals. However, genome-wide DNA demethylation occurs during early embryogenesis in both humans^11,12^ and mice^12,13^. Manually disturbing DNA methylome reprogramming via genetic knockout of DNA methyltransferases (DNMTs) or Tet3 in mice results in a failure of embryonic development^14–16^, indicating the importance of the DNA methylome in mammalian development.

Using whole-genome bisulphite sequencing (WGBS), it was shown that the DNA methylomes of mice are gradually demethylated after fertilization until the blastocyst stage^12,13^. Recently, the reduced representative bisulphite sequencing (RRBS) method has mainly been used to investigate the developmental process in humans^11,12^. Guo et al. also performed WGBS for inner cell mass (ICM) and post-implantation stages. Due to the nature of this method, the coverage of RRBS is limited to sections of the genome that are enriched with high-density CpGs, such as CpG islands (CGIs) and promoters^17^. Okae et al.^18^ profiled the DNA methylome of human oocytes and blastocysts using the post-bisulphite adapter-tagging (PBAT) method. Recently, the Tang laboratory^19^ performed single-cell DNA methylome sequencing of human preimplantation embryos with the PBAT method.

However, there are still several important questions that have not been answered. First, it has been found that the oocyte methylome is globally hypomethylated compared with the methylomes of sperm and somatic cells. However, it remains unknown whether the hypomethylated regions in oocytes are linked to any common genetic features. Second, it has been shown that promoter methylation is inversely correlated with gene expression, while gene body methylation is positively correlated with gene expression in oocytes^11^. However, the relationship between DNA methylation, CpG density and gene expression in oocytes has not been fully investigated. Third, it was shown in our previous study that the DNA methylation reprogramming of promoters is associated with embryonic development in both zebrafish and mice^9,13^. Nevertheless, the potential role of DNA methylation in regulating enhancer activity during reprogramming remains uninvestigated. Overall, the available research on the regulatory function of DNA methylation in human gametes and early embryogenesis remains limited.

## Results

### Conserved global dynamics of DNA methylome reprogramming during mammalian early embryogenesis

**Fig. 1:**
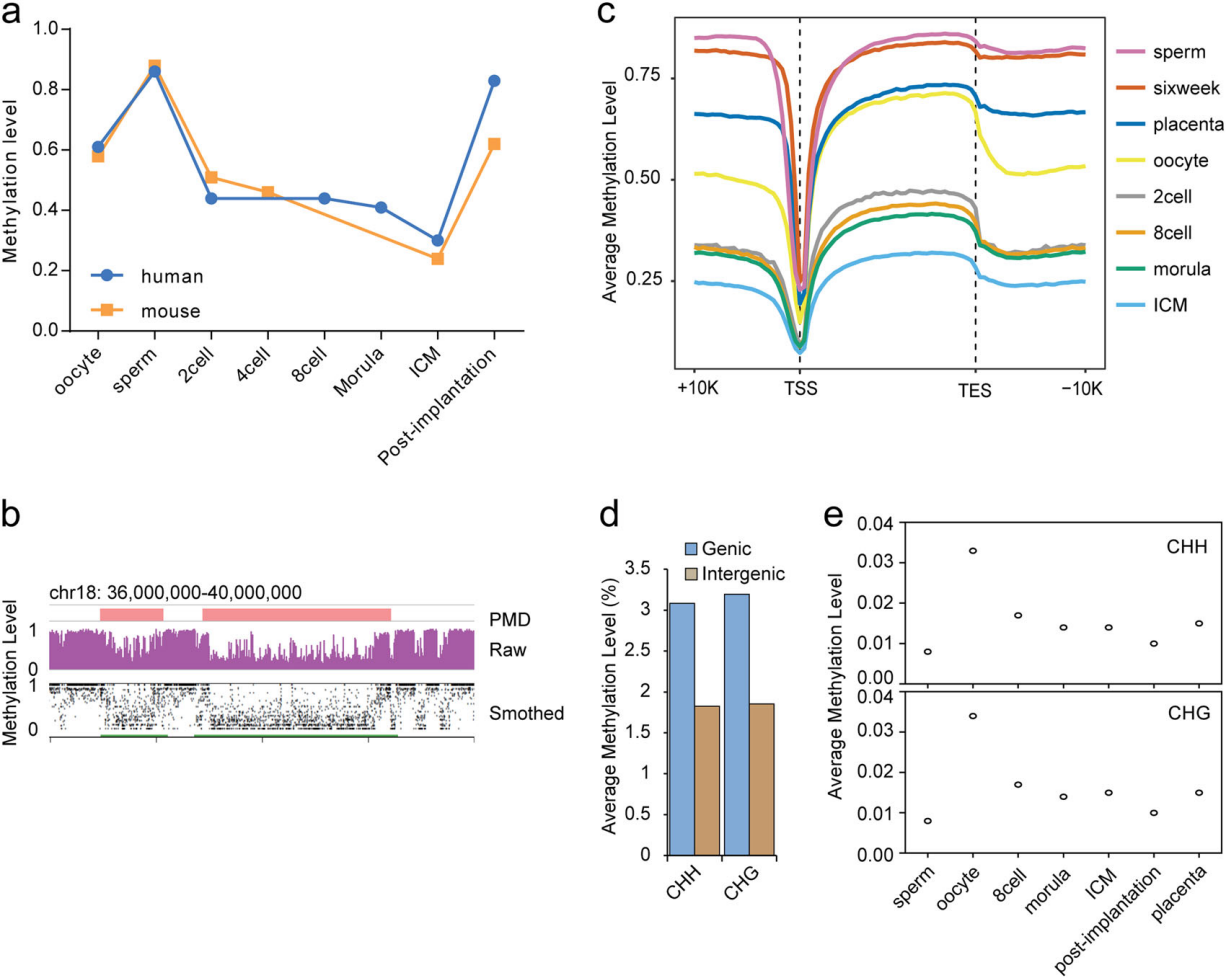
Dynamics of DNA methylation during mammalian early embryogenesis. **a** Average methylation level of genome-wide CpGs during mammalian early embryogenesis. The average methylation level in each stage is the mean of the methylation levels of each 500 bp tile. Only the tiles containing at least 3 CpG reads are considered. **b** Partially methylated domains (PMDs) in human oocytes. The upper panel is a snapshot of the genome browser for the PMDs. The lower panel is the DNA methylation smoothed by MethylseekR in the same region. Green bars indicate PMDs. **c** Distribution of methylation levels across all of the Refseq gene bodies, 10 kb upstream and downstream regions of transcriptional start sites (TSSs) and TESs (transcriptional end sites) for different stages. Gene bodies were divided into 40 intervals. The 10 kb upstream and downstream regions of TSSs and TSEs were divided into 25 intervals. The methylation levels of these intervals were calculated and plotted. Each tile should cover at least 5 CpG reads. **d** Genic and intergenic non-CpG methylation levels in human oocytes. **e** Dynamics of the average methylation levels of cytosines within CHG and CHH contents during early embryogenesis. The average methylation level is the mean value of the methylation levels of all non-CG cytosines. Only non-CG sites with a 5× depth were considered

Using our modified library generation method, we generated genome-wide DNA methylomes for human sperm, oocytes, 8-cell embryos, morula, ICM, and 6-week embryos as well as the full-term placenta at single-base resolution (Supplementary Table S1). As a result of the limited materials available, base-resolution 2-cell embryo DNA methylomes were generated via the published PBAT method^20^. We first plotted the distribution of methylation levels at CpG sites as well as CpG densities (Supplementary Fig. S1a-b). The methylation levels of CpG sites clearly showed a bimodal distribution in sperm and 6-week embryos. In contrast, the oocyte and placental methylomes exhibited many CpGs showing intermediate methylation. The data are consistent with the observations made in mice^8^. When we investigated the global dynamics of DNA methylomes during human early embryogenesis, our data showed that methylation levels declined from 0.61 to 0.30 during embryogenesis (Fig. 1a). The observed DNA reprogramming pattern was similar to the results from Tang’s laboratory^19^. Our data further showed that partially methylated domains could be detected in oocytes (Fig. 1b), and the methylation level in intergenic regions was much lower than that in genic regions at the oocyte stage (Fig. 1c).

Non-CpG cytosine methylation is observed in mammalian oocytes and neurons21. Accordingly, non-CpG methylation is observed in human oocytes. We found that the methylation level of non-CpGs in genic regions was higher than in intergenic regions (Fig. 1d). Our data also showed that the average non-CpG methylation level was much higher in oocytes than in other stages (Fig. 1e). Based on Fisher’s test, 11.68% of CHGs and 10.75% CHHs can be considered methylated cytosines, and the average methylation level of these methylated CHGs and CHHs in oocytes was approximately 0.3 (Supplementary Fig. S1c). In oocytes, non-CpG methylation was often found at CpA sites (Supplementary Fig. S1d), which is consistent with findings in neurons and mouse oocytes^21,22^.

### Oocyte-specific hypomethylated promoters, genic and intergenic regions

**Fig. 2:**
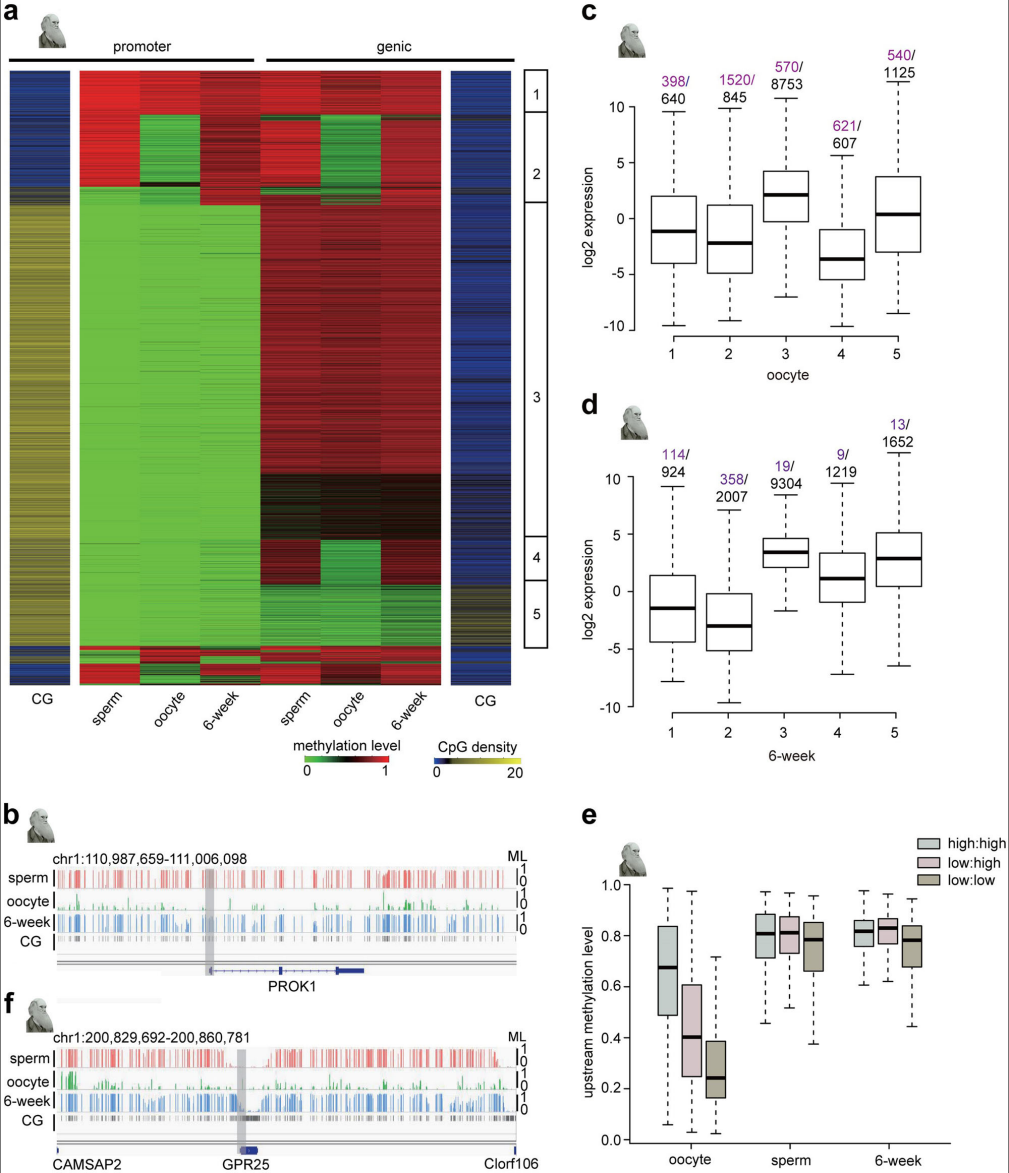
Oocyte-specific hypomethylated genic and intergenic regions. **a** The heat map represents the methylation level and CG density at all promoters and genic regions in human sperm, oocytes and 6-week embryos. Genes have been classified into five major groups according to the CG density and methylation patterns at the promoter and genic regions. **b** The graph represents the methylation level and CG density around *PROK1* gene regions. The promoter and genic region of the *PROK1* gene exhibit a low CG density. The methylation levels of the *PROK1* gene promoter and genic and intergenic regions are low in oocytes, but high in sperm and 6-week human embryos. **c** Gene expression levels in the 5 groups in oocytes. Purple numbers refer to the number of unexpressed genes. Black numbers refer to the number of expressed genes. The expression levels of the genes in group 2 were significantly lower than in groups 1, 3 and 5, and the expression levels of the genes in group 4 were significantly lower than in groups 1, 3 and 5. All *p* values of pair-wise comparisons were less than 0.001, and the *p* value was calculated based on the Mann–Whitney test. **d** Gene expression in the 5 groups in 6-week human embryos. Purple numbers refer to the number of unexpressed genes. Black numbers refer to the number of expressed genes. The expression levels of the genes in group 1 are significantly lower than in groups 3, 4 and 5, and the expression levels of the genes in group 2 are significantly lower than in groups 3, 4 and 5. All *p* values of pair-wise comparisons are less than 0.001, and the *p* value is calculated based on the Mann–Whitney test. **e** Intergenic methylation levels of genes with highly methylated promoters and genic regions (high: high), promoters with low methylation, but highly methylated genic regions (low: high), and promoters and genic regions with low methylation (low: low) in human sperm, oocytes and 6-week embryos. **f** The graph represents methylation levels and CG density around *GPR25* gene regions. The promoter and genic regions of the *GPR25* gene exhibit a high CG density and low methylation levels in sperm, oocytes and 6-week embryos. The methylation level of *GPR25* gene intergenic regions is low in oocytes, but high in sperm and 6-week embryos

The DNA methylation level in oocytes is much lower than in sperm and somatic cells. However, it is unclear which kinds of regions contribute to hypomethylation in oocytes. We plotted the DNA methylation levels of promoters and genic regions for each gene in human sperm, oocytes and embryos. Interestingly, oocyte-specific promoters generally exhibited low CpG densities (group 2 in Fig. 2a). Furthermore, oocyte-specific promoters with low CpG densities corresponded to oocyte-specific genic regions (group 2 in Fig. 2a, and Fig. 2b). In addition to these genic regions, a significant proportion of the genes with high-CpG-density promoters also presented oocyte-specific hypomethylated genic regions (group 4 in Fig. 2a), and these oocyte-specific hypomethylated genic regions generally exhibited a low CG density.

DNA methylation is involved in gene regulation. Therefore, we sought to analyze the association between DNA methylation and gene expression. For the oocyte transcriptomes of humans and mice, we used previously published data^13,23^, while for 6-week embryos, we generated an mRNA-Seq library. We found that genes with a high CpG density in genic regions were generally hypomethylated in sperm, oocytes and 6-week embryos and showed expression in oocytes (group 5 in Fig. 2c and Supplementary Fig. S2a). This result was different from previous findings indicating that gene expression was positively correlated with the methylation level in genic regions^11^. However, genes with a low CpG density and hypomethylated genic regions did not show any expression, and the remaining genes exhibited low expression levels in the oocytes of humans (groups 2 and 4 in Fig. 2a and Fig. 2c, *p* < 0.001) and mice (groups 2 and 4 in Supplementary Fig. S2a, *p* < 0.001), although these gene promoters were hypomethylated. In contrast, genes with hypermethylated promoters showed low expression levels in human 6-week embryos (groups 1 and 2 in Fig. 2a, d, *p* < 0.001) and mouse E7.5 embryos (groups 1 and 2 in Supplementary Fig. S2b, *p* < 0.001). Taken together, the results indicated that hypermethylated promoters correlated with low gene expression in embryos, while hypomethylated genic regions with a low CG density, correlated with gene silencing in oocytes.

We further compared the methylation levels of intergenic regions between oocytes and sperm. Interestingly, intergenic regions presented low methylation if both the promoters and genic regions presented low methylation, while the intergenic regions were middle methylated if only the promoters showed low methylation in the oocytes of humans (Fig. 2e, f and Supplementary Fig. S2c) and mice (Supplementary Fig. S2d). In contrast, intergenic regions were generally highly methylated regardless of whether the promoters were highly methylated or showed low methylation in human sperm and 6-week embryos (Fig. 2e, f ) and mouse sperm and E7.5 embryos (Supplementary Fig. S2d). In summary, hypomethylated oocyte promoters define oocyte-specific hypomethylated genic and intergenic regions, which contribute to the hypomethylation pattern of mammalian oocytes.

### Dynamics of repeat elements during early embryogenesis

**Fig. 3:**
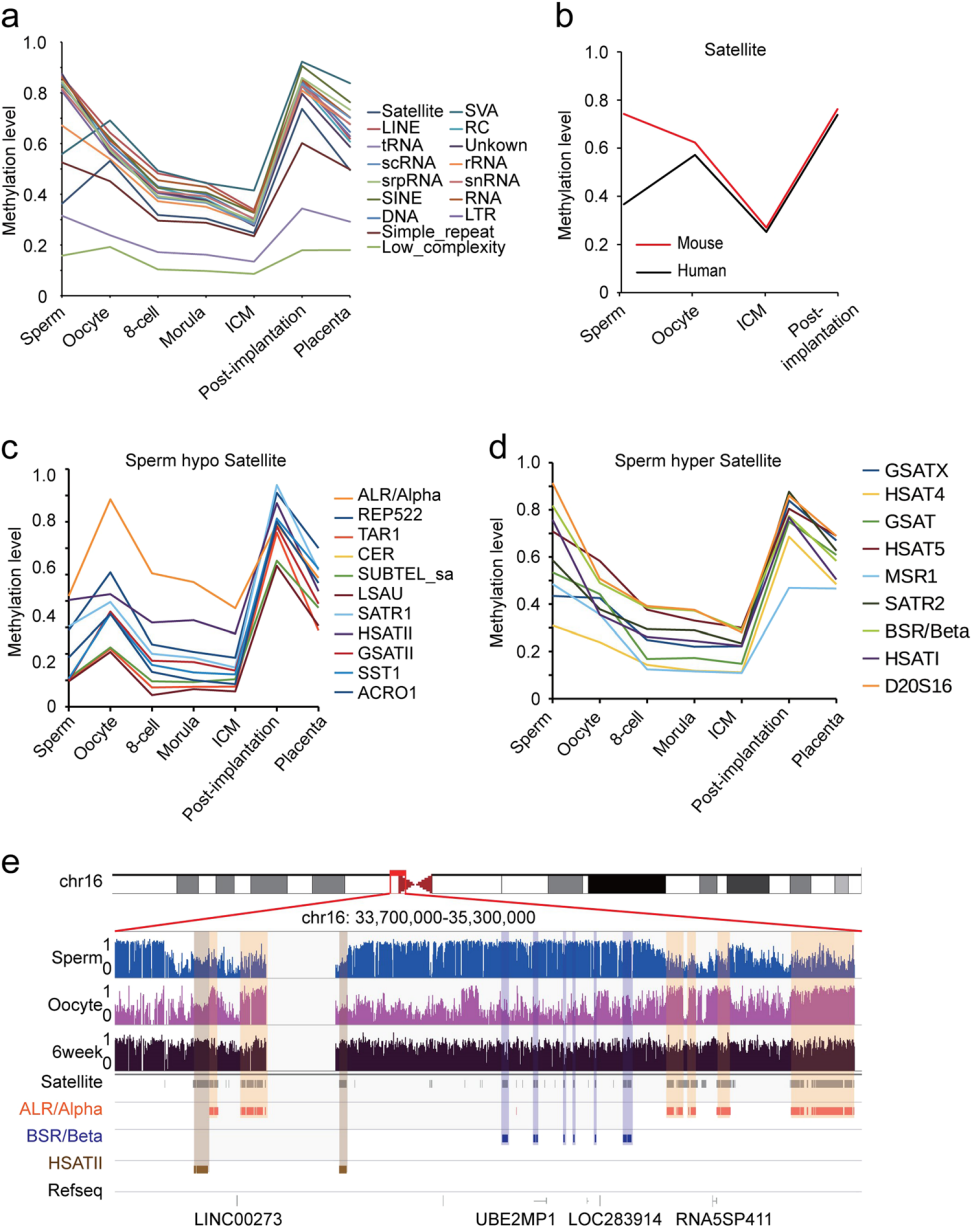
Dynamics of DNA methylation in repeat elements. **a** Dynamics of the methylation levels of various repeat elements. **b** Comparison of the methylation dynamics of satellite repeats between humans and mice. **c** Dynamics of the methylation levels of satellite repeat subfamilies that are oocyte specific and highly methylated during early embryogenesis. **d** Dynamics of the methylation levels of satellite repeat subfamilies that are sperm specific and highly methylated during early embryogenesis. **e** Graphical representation of a genomic region around the centromere of human chromosome 16 showing the methylation levels of different subfamilies of satellite repeats in sperm, oocytes and 6-week embryos

Next, we explored the methylation dynamics of repeat elements. Our data showed that most repeat elements in the sperm were hypermethylated compared with those in the oocytes, except for low-complexity repeats and satellites (Fig. 3a). The average methylation level of the satellites in human oocytes was higher than in human sperm, which is opposite the pattern observed in mouse gametes (Fig. 3b). Satellite repeats are mainly located in centromeres and telomeres. Centromere satellite repeats are implicated in facilitating meiotic and mitotic chromosomal segregation^24,25^. Several subfamilies of centromere repeats are hypomethylated in sperm compared with oocytes (Fig. 3c), including *ALR/Alpha*, which is the major DNA component of centromeres in primates, whereas the *BSR/Beta* subfamily is hypermethylated in sperm (Fig. 3d). It was also observed that the methylation state of the *HSATII* subfamily was similar between sperm and oocytes (Fig. 3c). Figure 3e shows a representative centromere region containing the *BSR/Beta*, *ALR/alpha* and *HSATII* satellite subfamilies.

### Methylation reprogramming of promoters is associated with development

We further investigated the dynamics of other functional genomic elements during early human embryogenesis. Our data showed that most functional elements were significantly demethylated (Supplementary Fig. S3a). The distribution of the CpG methylation level in exons, lincRNA, miRNA and pseudogene regions showed a highly conserved pattern in humans and mice (Supplementary Fig. S3b-e). The average methylation level of miRNAs was lower than in exons, lincRNA and pseudogenes in gametes and 6-week/E7.5 embryos (Supplementary Fig. S3e). A bimodal pattern of miRNA methylation could be observed in sperm and 6-week/E7.5 embryos, but not in oocytes (Supplementary Fig. S3e).

**Fig. 4:**
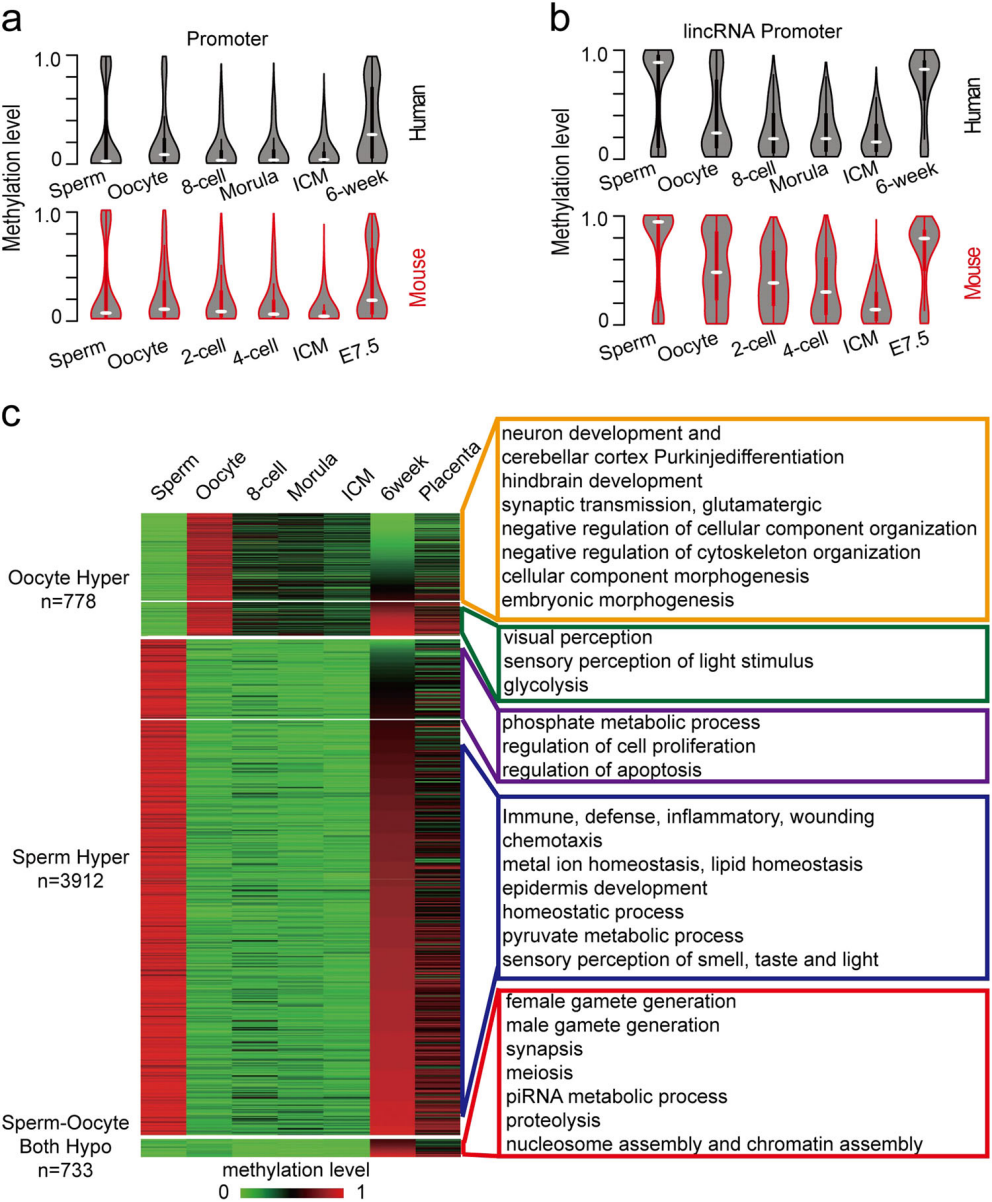
Dynamics of DNA methylation at promoters. **a** Violin plots of the dynamics for promoters of humans and mice during early embryogenesis. **b** Violin plots of the dynamics for lincRNA promoters in humans and mice during early embryogenesis. **c** Heat map of the methylation reprogramming of promoters from human gametes to early embryos. Genes with reprogrammed promoters during early embryogenesis were used for DAVID GO enrichment analyses. GO biological pathways with a *p* value <0.05 were considered to show significant enrichment

DNA methylation in promoters plays a critical role in gene regulation, but the understanding of how these elements function in human early development is still poor. The distribution of methylation levels at the promoters of both protein-coding genes and lincRNAs (Fig. 4a, b) shows a conserved pattern between humans and mice. However, in contrast to the methylation of the promoters of protein-coding genes, lincRNA promoters are hypermethylated in sperm and 6-week/E7.5 embryos^26^. Next, we investigated how DNA methylation regulates gene expression using previously published transcriptome data^23^. Our analysis showed that gene expression at different developmental stages was inversely correlated with the DNA methylation level at promoters (Supplementary Fig. S3f).

In both zebrafish and mice, the methylation dynamics of promoters are associated with embryonic development^9,12,13^. The association of promoter reprogramming in early human embryogenesis has not previously been investigated. We focused on the analysis of the gamete-specific methylated promoters and performed gene ontology (GO) analyses using a list of genes with differentially methylated promoters (Supplementary Table S2). Genes with promoters that were hypomethylated in sperm and 6-week-old embryos, but hypermethylated in oocytes, were enriched in nervous system development and embryonic development (Fig. 4c orange box). Genes with promoters that were hypermethylated in sperm and 6-week-old embryos and hypomethylated in oocytes were enriched in the immune response pathway and ion homoeostasis (Fig. 4c blue box). These results are consistent with previous studies of early mouse embryogenesis^13^. Our data also showed that genes exhibiting hypomethylated promoters in sperm and oocytes but hypermethylated promoters in 6-week-old embryos were enriched in male and female gametes generation, meiosis, and piRNA pathways (Fig. 4c red box), which were unique to sperm and oocyte development.

### Methylation reprogramming of enhancers orchestrates embryonic development in mammals

**Fig. 5:**
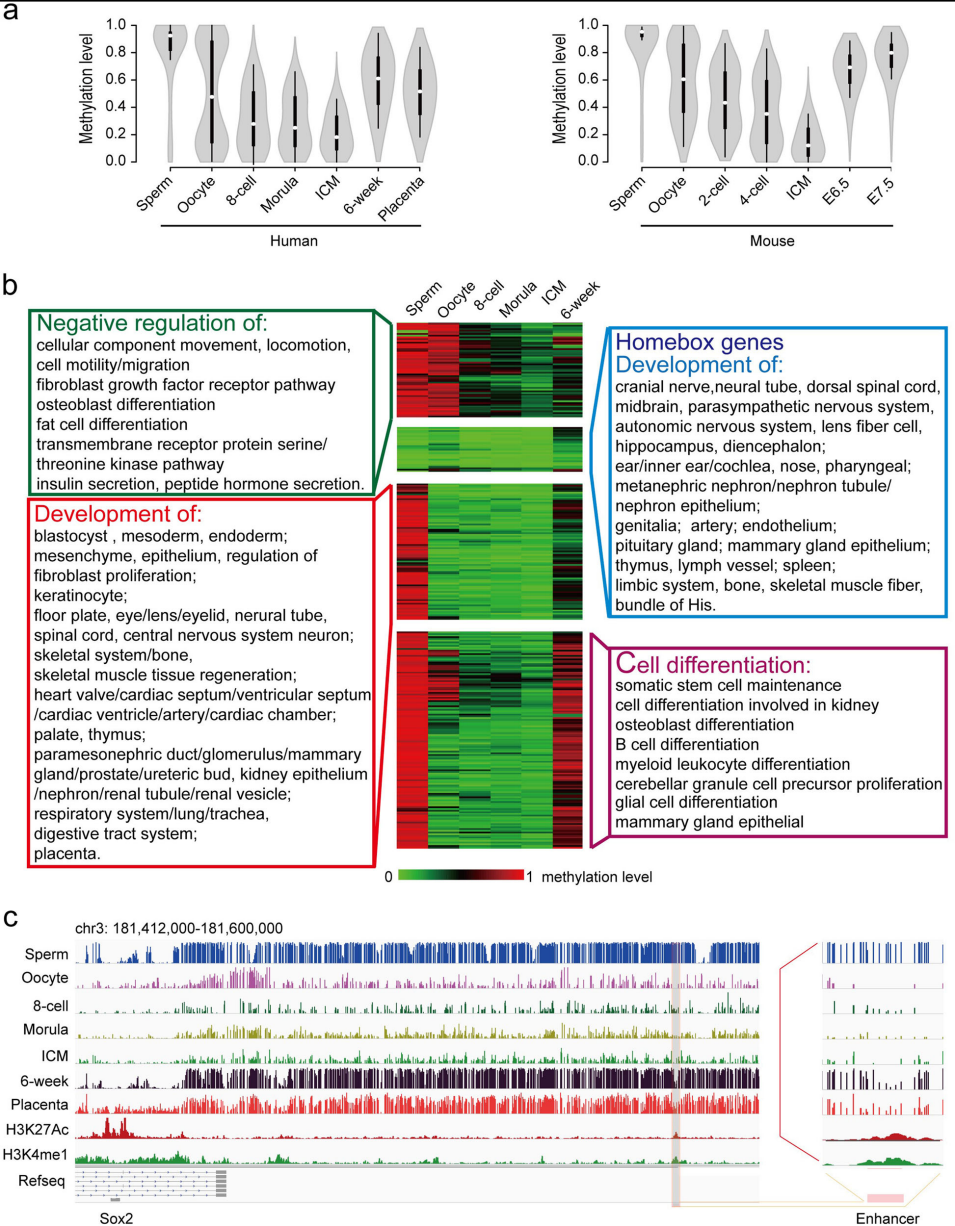
Dynamics of DNA methylation at enhancers. **a** Violin plots of the dynamics for enhancers in humans and mice during early embryogenesis. **b** Heat map of the methylation reprogramming of enhancers from human gametes to early embryos. An unsupervised hierarchical cluster analysis was performed, and then GO enrichment analysis of each group was performed with GREAT online tools. **c** Graphical representations of the methylation status of distal enhancers of *SOX2* in human gametes and early embryos. The H3K27ac and H3K4me1 datasets came from H1 cells generated by the NIH Roadmap project (the accession numbers are GSM466732 and GSM409307)

Enhancers are important cis-regulatory elements that are highly associated with tissue-specific gene expression^27,28^. DNA methylation of enhancers is an important mechanism regarding the binding of transcription factors and regulation of transcription^29^. Due to the limited coverage of RRBS, the understanding of the potential roles of enhancers during human embryogenesis is very limited. Here, we investigated the methylation dynamics of enhancers. Due to the lack of an enhancer database for gametes and early embryos, the putative enhancers in human embryonic stem cells (hESCs) and mouse ESCs (mESCs) were used instead^30,31^. Enhancers were generally highly methylated in sperm whereas they exhibited a bimodal distribution in oocytes, which was conserved between humans and mice (Fig. 5a, b, Supplementary Fig. S4, and Table S3). Further GO analyses showed that genes with enhancers that were hypermethylated in gametes, but hypomethylated in 6-week embryos were highly enriched in cellular component movement, locomotion and migration (Fig. 5b green box and Supplementary Table S4). Additionally, genes with enhancers that were hypomethylated in oocytes and 6-week embryos were highly enriched in multiple human organs (Fig. 5b blue and red panel), and genes with enhancers that were hypermethylated in 6-week embryos were highly enriched in cell differentiation (Fig. 5b purple panel). An example of the distal enhancer of the *Sox2* gene^31^ is presented in Fig. 5c. In mice, the enhancer methylation pattern also displayed a conserved regulatory function (Supplementary Fig. S1 and Table S5). Furthermore, gene expression was inversely correlated with the DNA methylation level in enhancers (Supplementary Fig. S3g). Our results suggest that the methylation reprogramming of enhancers may facilitate the temporal control of embryonic development and organogenesis.

### Genomic imprinting in mammals

Genomic imprinting plays an important role in mammalian embryogenesis^32^. Imprinting disorders can result in human pathologies such as Prader-Willi syndrome and Angelman syndrome^33^. Approximately 100 genes are reported to be imprinted in humans^34^, but the methylation states of their imprinting control regions (ICRs) are not well validated, especially in oocytes and early embryos. According to the methylation states of gametes, early embryos, placentas and human skin tissue (Roadmap dataset), 24 regions were confirmed as germ-line ICRs in humans (Supplementary Table S6 and Figure S5a), 16 of which were conserved between humans and mice, while 8 were human-specific ICRs. Additionally, 20 of these germ-line ICRs were also identified as ICRs in placenta (Supplementary Fig. S5a). In addition, there were 9 placenta-specific ICRs in humans (Supplementary Fig. S5a). Two of the ICRs that were tracked with paired-end reads are shown in Supplementary Fig. S5a and S5c.

ICRs often contain CGIs. We found that 30 ICRs were related to CGIs (Supplementary Fig. S3h). Most ICRs are maternally imprinted. Accordingly, there were more oocyte-specific methylated CGIs than sperm-specific methylated CGIs in both humans (Supplementary Fig. S3h) and mice, which differs from the pattern observed for other genomic elements. We also found that the methylated CGIs in gametes were mainly located in introns (Supplementary Fig. S3h, pie chart).

## Discussion

Here, we generated genome-wide methylomes for human early embryos using modified library generation methods at single-base resolution. We further compared human methylomes with mouse methylomes at gametic and embryonic stages. We found that oocyte-specific hypomethylated promoters generally exhibited low CpG densities (Fig. 2a). Moreover, genes with oocyte-specific hypomethylated promoters generally showed oocyte-specific hypomethylated genic and intergenic regions, and these hypomethylated regions contributed to the hypomethylation pattern of mammalian oocytes (Fig. 2a). Our data showed that intergenic regions presented low methylation if both promoters and genic regions displayed low methylation, while intergenic regions were moderately methylated if only promoters exhibited low methylation in the oocytes of humans (Fig. 2e, f and Supplementary Fig. S2c) and mice (Supplementary Fig. S2d). In contrast, intergenic regions were generally highly methylated regardless of whether the promoters were highly methylated, but showed low methylation in human sperm and H6W embryos (Fig. 2e, f) and mouse sperm and E7.5 embryos (Supplementary Fig. S2d). In summary, hypomethylated oocyte promoters define oocyte-specific hypomethylated genic and intergenic regions, which contribute to the hypomethylation pattern of mammalian oocytes. All previous studies have indicated that the methylation level of genic regions is positively associated with gene expression. We showed that hypomethylated genic regions with a low CG density correlate with gene silencing in oocytes. In contrast, hypomethylated genic regions with a high CG density correspond to significant gene expression in oocytes, which contrasts with previous findings regarding the association between genic methylation and gene expression. We further demonstrated that compared with promoters, enhancers are more dynamically reprogrammed by DNA methylation to orchestrate the elaborate processes of embryogenesis and organogenesis. More importantly, our data show that the methylation reprogramming of enhancers during early embryogenesis is highly associated with the development of almost all human organs.

Our previous studies demonstrated that the sperm DNA methylome is inherited by the zebrafish early embryo^9^ and that active demethylation occurs in the both maternal and paternal genomes of mammalian early embryos^13,35^. Although RRBS^11^ has been used to study DNA methylation reprogramming in humans, the coverage is limited to sections of the genome enriched with high-density CpG regions, which limits our understanding of the global dynamics and the regulatory function of DNA methylation in gametes and early embryogenesis. Recently, Zhu et al. performed single-cell PBAT DNA methylome sequencing of human preimplantation embryos and revealed that three waves of global demethylation occurred in preimplantation embryos^19^. This finding correlates with our results (Fig. 1a).

The DNA methylome of mammalian oocytes is known to be hypomethylated compared with that of sperm and somatic cells. However, it is still unknown whether the hypomethylated regions are linked to any common genetic features. In addition, the association between DNA methylation and gene expression in oocytes has not been fully investigated. We found that hypomethylated promoter patterns define hypomethylated genic and intergenic patterns in oocytes. Furthermore, hypomethylated genic regions with low CG densities correlate with gene silencing in oocytes, whereas hypomethylated promoters correlate with high gene expression in embryos. It appears that there is a unique mechanism involved in establishing the DNA methylome in oocytes, which is different from that in sperm and early embryos.

Several studies have shown a positive correlation between gene expression and the DNA methylation level of genic regions^11^. However, our data indicated that this is only the case if genic regions exhibit a low CpG density. In genes with high-CpG-density genic regions, hypomethylated genic regions correspond to high gene expression. Therefore, we argue that the methylation level of genic regions may not be involved in the regulation of gene expression.

Genome-wide demethylation occurs during mammalian development. Some regions, such as ICRs, are protected from the demethylation machinery. However, our knowledge regarding how these sequences are protected from demethylation as well as the selection mechanism determining which regions are protected from demethylation is still limited. Genome-wide re-methylation occurs after embryo implantation in mammals. However, the initiation mechanism of this global reprogramming remains unknown.

Our data provide a valuable resource for future research on human embryonic development. Moreover, they show that oocyte-specific regions are linked to the CpG density of promoters. Hypomethylated genic regions, but not hypermethylated promoters, are associated with gene silencing in oocytes. Our data suggest that DNA methylation reprogramming plays important roles in mammalian development.

## Materials and Methods

### Sample collection

The human tissue collection procedure and study protocol employed in this research were approved by the Institutional Review Board of Peking University Third Hospital (Research license 2012SZ015). The methods closely followed the guidelines legislated and posted by the Ministry of Health of the People’s Republic of China. The patients were informed of all details of the procedure, including sample utility and research destination. The patients voluntarily signed an informed consent document. Human embryos at the blastocyst stages were donated by couples who had conceived at least one healthy baby through assisted reproductive technology (ART) treatment. These donor couples, whose infertility was purely due to female tubal factors, had already given birth to a healthy baby through an IVF cycle. They then donated their surplus frozen embryos for research, signing written informed consent forms. The embryos were then graded according to the Gardner morphological blastocyst grading system before collection for further methylation analyses.

All of the embryos used in this study were of good quality. For the oocyte methylome, two replicates were evaluated, with approximately 20 oocytes at the MII stage in each replicate. For the 2-cell methylome, there was only one replicate because we did not obtain additional 2-cell embryos for methylome analysis. We used five 2-cell embryos to construct the library. Two replicates of both the 8-cell and morula methylomes were also examined, where ten 8-cell embryos or 5 morula embryos were used for each replicate. Two replicates were included for the ICM methylome as well, where 5 ICMs were used for each replicate. For the sperm and 6-week embryo methylomes, 3 replicates were included. The sperm samples came from 3 individual donors.

### MethylC-Seq library generation

DNA methylation libraries of human MII oocytes, 8-cell embryos, morulas, and inner cell masses were constructed with our modified library generation method. Briefly, approximately 20 oocytes, ten 8-cell embryos, 5 morula-stage embryos or 5 ICM embryos were lysed in 5 µL of lysis buffer (20 mM Tris, 2 mM EDTA, 20 mM KCl, 1 mg/mL protease) for 1.5 h at 56 °C and then heat inactivated for 30 min at 75 °C. Next, 45 μL of nuclease-free water and 0.5% spike-in unmethylated lambda DNA (Promega) were added to the lysate, and the DNA was fragmented with a Covaris S2 ultrasonicator. The shearing conditions were as follows: 5% duty cycle, intensity of 5, 200 cycles/burst and duration of 60 s. The lysate was then concentrated to 30 µL. The fragmented DNA was end-repaired via incubation with 5 µL of end-repair enzyme mixture (3.5 µL T4 DNA ligase buffer (NEB), 0.35 µL 10 mM dNTPs, 1.15 µL NEBNext End Repair Enzyme Mix (NEB)) for 30 min at 20 °C, followed by heat inactivation for 30 min at 75 °C. After end-repair, 5 µL of dA-tailing mixture (0.5 µL T4 DNA ligase buffer, 1 µL Klenow exo- (NEB), 0.5 µL 100 mM dATP and 3 µL nuclease-free water) was added to the tube, followed by incubation for 30 min at 37 °C and heat inactivation for 30 min at 75 °C. Finally, 10 µL of ligation mixture (1 µL T4 DNA ligase buffer, 0.5 µL 100 mM ATP, 1.5 µL 50 mM cytosine-methylated Illumina adapter, 2 µL T4 DNA ligase (NEB) and 5 µL nuclease-free water) was added to the tube, followed by incubation at 16 °C overnight. Next, 100 ng of Carrier RNA was added to the tube, and a bisulphite conversion reaction was performed with the EZ DNA methylation-Gold Kit (Zymo Research) according to the manufacturer’s instructions. The purified DNA was amplified through 6 cycles of PCR using KAPA HiFi HotStart Uracil + ReadyMix (KAPA). The amplified DNA was subsequently purified with Ampure XP beads (Beckman) to removed short fragments and adapter self-ligations. Then, another round of 6–8 cycles of PCR was performed to obtain sufficient molecules for sequencing. The DNA methylome libraries were sequenced on the HiSeq2000 or HiSeq2500 platform. At least two biological replicates were included for each developmental stage. Then, 2-cell embryos were constructed via the PBAT method^20^.

### MethylC-Seq: read filtering, alignment, quantification of methylation levels

Reads were trimmed using Trimmomatic^36^ with default parameters to remove reads containing adapters and reads of low quality. The trimmed reads were aligned using Bismark (V12.5)^37^. Bisulphite Read Mapper was employed against the human reference hg19 with stringent parameters: -N 1 -X 600. After alignment, PCR duplications were removed with Picard (http://broadinstitute.github.io/picard/). Overlapping regions among the uniquely mapped paired reads were clipped using the clipOverlap function of BamUtil (http://genome.sph.umich.edu/wiki/BamUtil:_clipOverlap). CpG and non-CpG methylation levels were extracted with the mpileup function of SAMtools (v0.1.19)^38^. Strands were then merged to calculate the CpG methylation level per site. The average methylation level in each stage was the mean of the methylation levels of each 500 bp tile. Because of the limited materials (the 2-cell stage samples only contained 10 cells), the coverage of the 2-cell stage is low. When the analyses included the 2-cell stage, we used 500 bp tiles as bins to calculate the average methylation level. Each tile should be covered at least 3 times. As for other analyses, CpG sites with at least 3× coverage were considered.

### Annotation datasets

All of the datasets generated and used here were based on the hg19 (GRCh37) reference. The datasets for human and mouse enhancers were obtained from Bing Ren’s laboratory^30,39^. Refseq, RepeatMasker and other tracks used in this study were downloaded from the UCSC genome browser.

### Differentially methylated regions

For DMR analyses, we used the R package bsseq, which is a smoothing local likelihood method that produces precise results, even for low coverage data, in addition to exhibiting the ability to handle biological replicates^40^. DMRs containing at least 5 CpGs where the difference between two groups was higher than 0.2 were used for further analyses.

### Differentially methylated enhancers

Since enhancer profiling data were not available for human and mouse gametes and early embryos, we used the data of mESC and hESC as given in refs. ^30,39^. We expanded the predicted enhancer peaks in the reference in both the upstream and downstream direction for 500 bp. Only enhancers containing at least 5 CpGs that were covered by at least 25 reads were considered for further analysis. Functional analysis of enhancers was performed using the GREAT tool^41^ with the default settings (5+1 kb basal, up to 1 Mb extension) for proximal and distal binding events. Binomial enriched terms were significant at a false discovery rate of 0.05 by the hypergeometric test.

### Differentially methylated promoters

Promoters are defined as the regions 1 kb upstream from the transcriptional start sites (TSSs) of human Refseq transcripts (h19). Only promoters containing at least 5 CpGs and covered by at least 25 reads were considered for further analysis. The methylation level of each promoter was calculated as the ratio of the number of methylated Cs to all of the methylated and unmethylated Cs in the promoter. Promoters showing a methylation level ≥0.75 in oocytes and ≤0.25 in sperm were classified as oocyte-specific highly methylated promoters, whereas sperm-specific highly methylated promoters were defined based on a methylation level ≥0.75 in sperm and ≤0.25 in oocytes. These gamete-specific highly methylated promoters were subjected to GO analyses according to their methylation status in 6-week embryos. GO analysis of genes with differentially methylated promoters was performed using DAVID^42^. GO terms with a *p* value of <0.05 were considered statistically significant.

### mRNA-Seq library generation and data analysis

Six-week foetal RNA was extracted with the Direct-zol™ RNA MiniPrep kit (Zymo). RNA-Seq libraries were constructed with the NEBNext® Ultra™ RNA Library Prep Kit for Illumina (NEB) according to the manufacturer’s instructions. QC-passed libraries were sequenced on the HiSeq2500 platform with the paired-end module. Adapter-containing and low-quality reads were trimmed, followed by alignment with TopHat. The unique reads were used to calculate the fragments per kilobase of exon per million fragments mapped (FPKM) with Cufflinks v2.0.2 (http://cufflinks.cbcb.umd.edu).

### External data used in this study

**Table:** 

Resource	Source	Identifier
Human oocyte transcriptome	Yan et al., 2013	GSE36552
Mouse transcriptome and methylome	Wang et al., 2014	GSE56697
Histone data	NIH Roadmap project	GSM466732, GSM409307

### Accession codes

Sequencing data have been deposited in the Genome Sequence Archive (GSA) under project number CRA000114.

## Electronic supplementary material

Supplementary Figures

Table S1

Table S2

Table S3

Table S4

Table S5

Table S6

## Electronic supplementary material

**Supplementary Information** accompanies the paper at (10.1038/s41421-018-0039-9).
