# Supplementary material for: DNA methylation reprogramming of functional elements during mammalian embryonic development
Source: Cell Discov. 2018 Aug 7;4:41. doi: 10.1038/s41421-018-0039-9 (PMC6079081; doi:10.1038/s41421-018-0039-9)
Supplement: Supplementary file 1 — Supplementary Figures [file 41421_2018_39_MOESM7_ESM.docx]

**
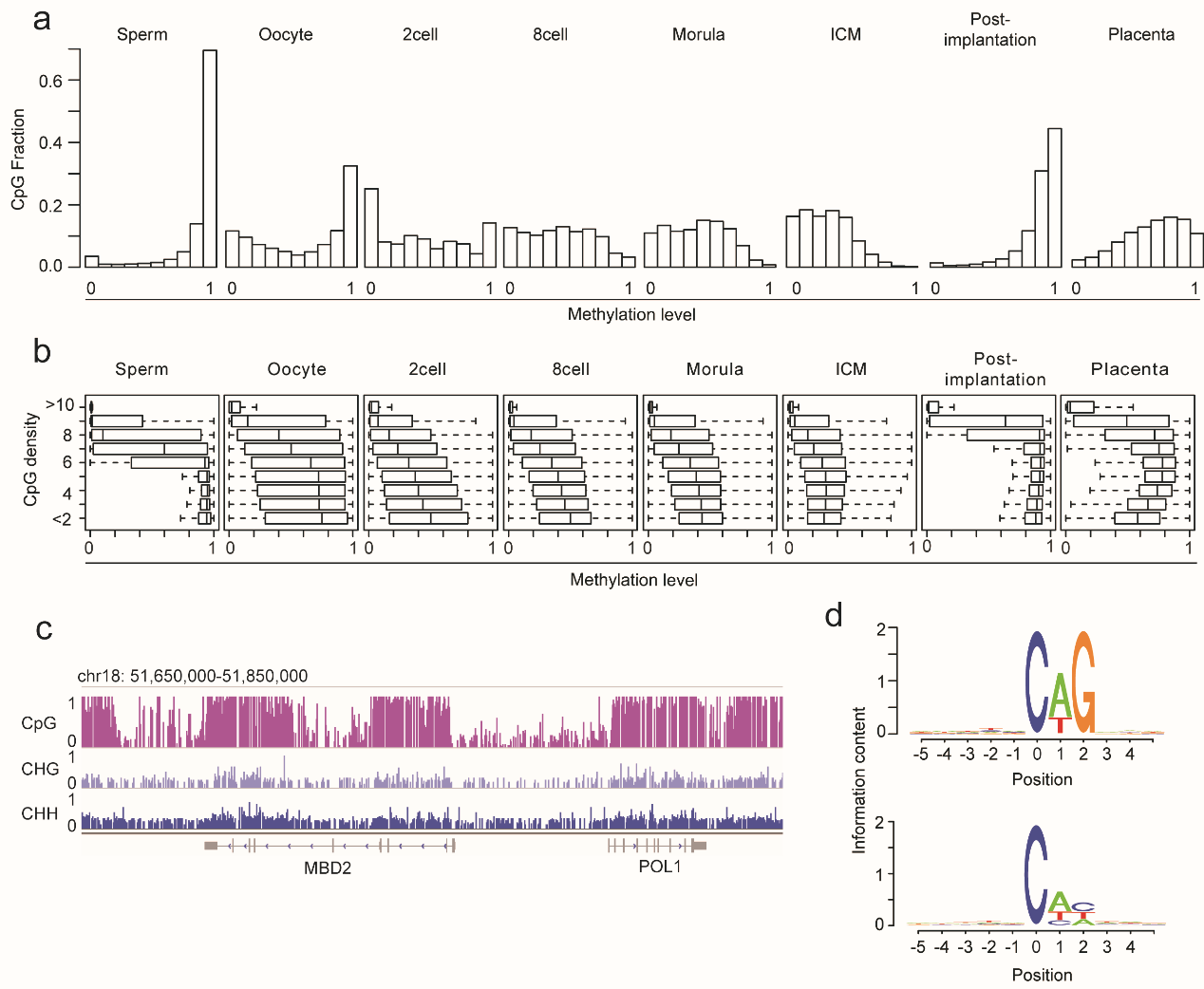
**

**Figure S1** Dynamics of DNA methylation during mammalian early embryogenesis. **(a)** Fraction of all 500 bp tiles with different methylation levels at different stages in humans. Each tile should cover at least 3 CpG reads. **(b)** Boxplots of methylation levels at different CpG densities for 500 bp tiles in human. The bullseye indicates the median, and boxes and lines indicate the 25th/75th and 2.5th/97.5th percentiles, respectively. The CpG density for a CpG site is the number of CpG sites in its 50 bp up- and downstream regions. The CpG density for a 500 bp tile is the average CpG density of all CpG sites in the tile. Only tiles containing 3 CpG reads are considered. **(c)** A snapshot of non-CpG methylation in human oocytes. **(d)** Motif analyses of non-CpG methylated sites in human oocytes.

**
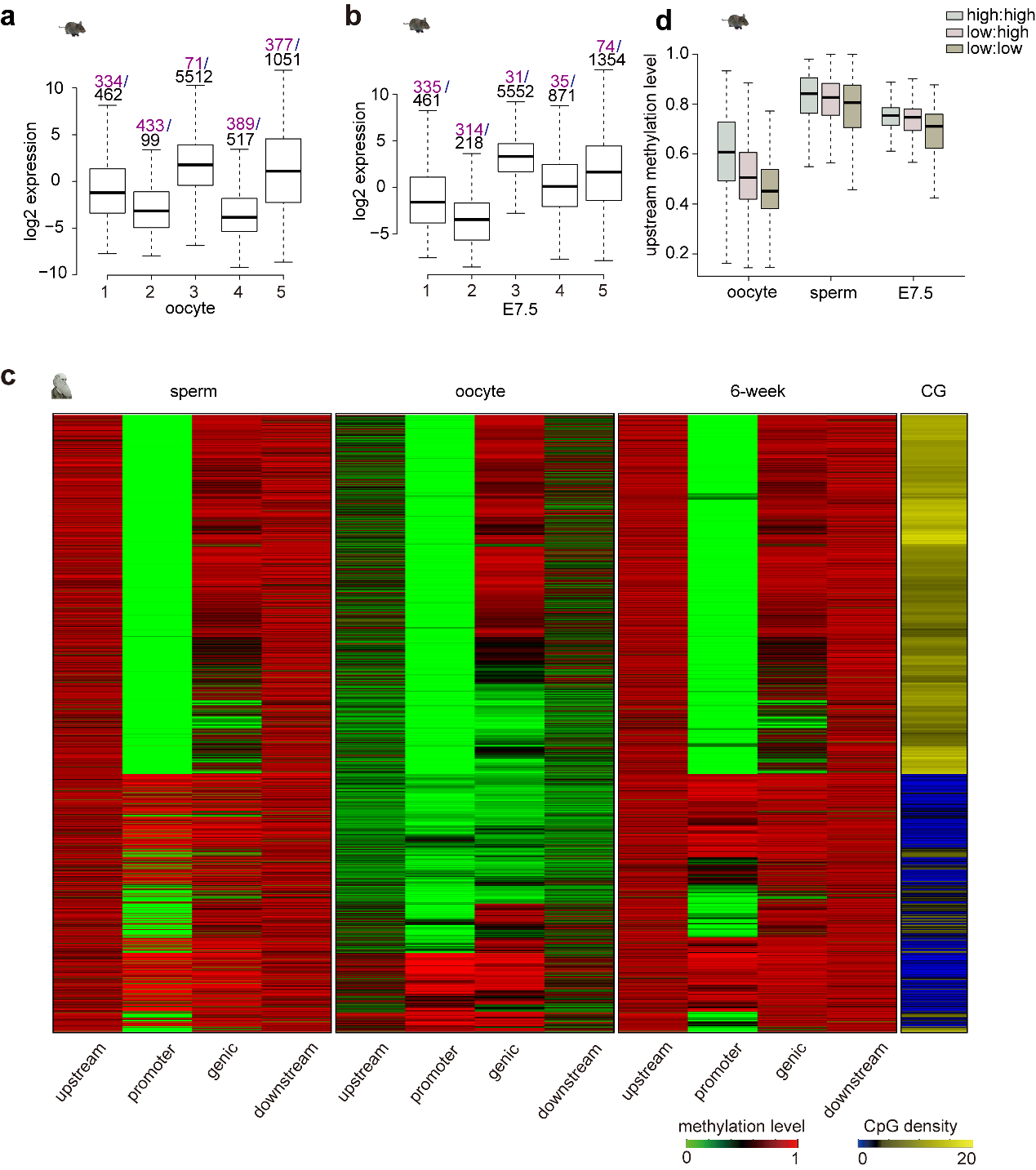
**

**Figure S2** Hypomethylated genic regions and intergenic regions in mammalian oocytes, related to Figure 2. **(a)** Gene expression in the 5 groups in mouse oocytes. Purple numbers refer to number of un-expressed genes. Black numbers refer to the number of expressed genes. The expression levels of the genes in group 2 are significantly lower than in groups 1, 3, and 5, and the expression levels of the genes in group 4 are significantly lower than in groups 1, 3, and 5. All *p* values of pair-wise comparisons are less than 0.001, and the *p* value is calculated based on the Mann-Whitney test. **(b)** Gene expression in the 5 groups in mouse E7.5 embryos. Purple numbers refer to the number of un-expressed genes. Black numbers refer to the number of expressed genes. The expression levels of the genes in group 1 are significantly lower than in groups 3, 4, and 5, and the expression levels of the genes in group 2 are significantly lower than in groups 3, 4, and 5. All *p* values of pair-wise comparisons are less than 0.001, and the *p* value is calculated based on the Mann-Whitney test. **(c)** The heat map represents the methylation level and CG density of all promoters, genic regions and intergenic regions in human sperm, oocytes and 6-week embryos. “CG” refers to the promoter CG density. **(d)** Intergenic methylation levels of the genes with highly methylated promoters and genic regions (high high), promoters with low methylation/highly methylated genic regions (low high), and promoters and genic regions with low methylation (low low) in mouse sperm, oocytes and E7.5 embryos.


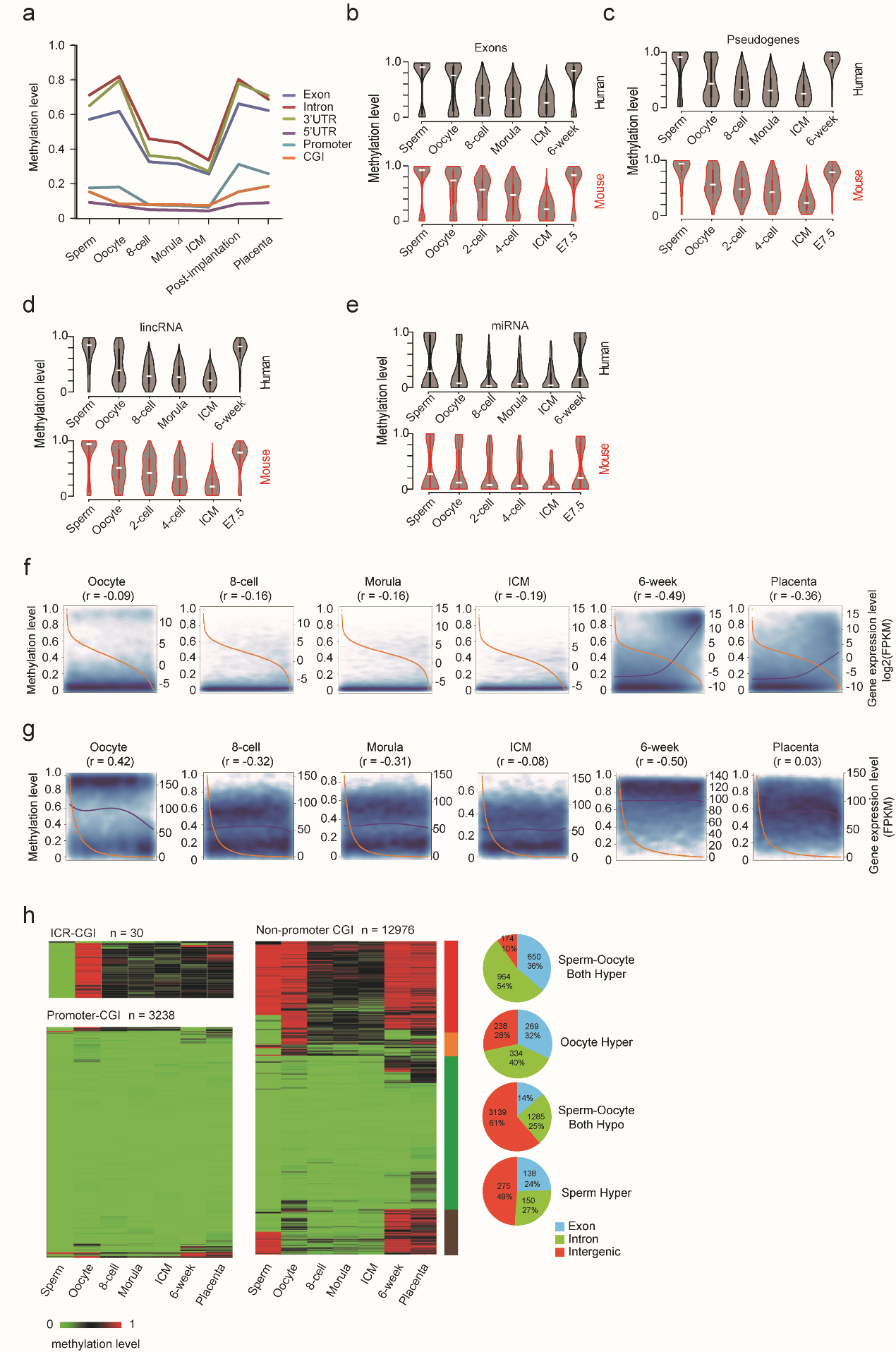


**Figure S3** Methylation reprogramming of functional genomic elements, related to Figure 4. **(a)** Dynamics of the methylation levels of different genic-related genomic elements during early embryogenesis. **(b-e)** Violin plots of the dynamics for exons, pseudogenes, lincRNAs and miRNAs in humans and mice during early embryogenesis, indicating the conserved distribution and dynamic trends in mammals. **(f)** Inverse correlations between gene expression and DNA methylation levels in promoter regions (1 kb upstream of TSS). The promoter methylation level is shown as smoothed blue scatter, while the gene expression level (log2 (FPKM)) is sorted and plotted as an orange line. Pearson correlation coefficients (*r*) were calculated between the gene expression levels and DNA methylation levels of promoters. **(g)** Inverse correlations between gene expression and DNA methylation levels in enhancer regions. **(h)** Dynamics of the methylation levels of all CGIs during early embryogenesis.


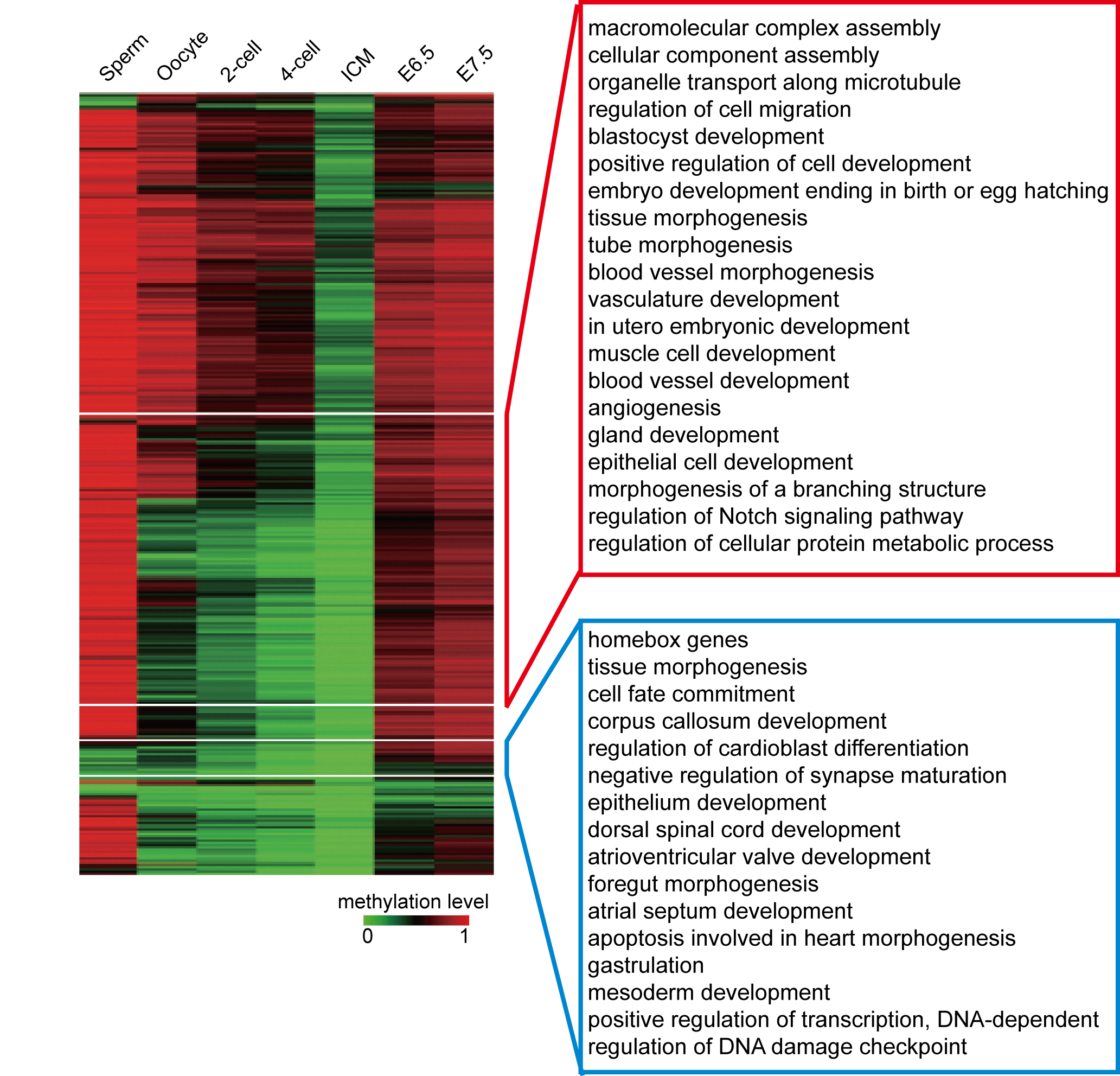


**Figure S4** Dynamics of DNA methylation in mouse enhancers, related to Figure 5. Heat map of the methylation reprogramming of enhancers from mouse gametes to early embryos. Hierarchical cluster analysis was performed according to the dynamic trend of DNA methylation. GO enrichment for each cluster was analysed using GREAT tools with default association settings.

**
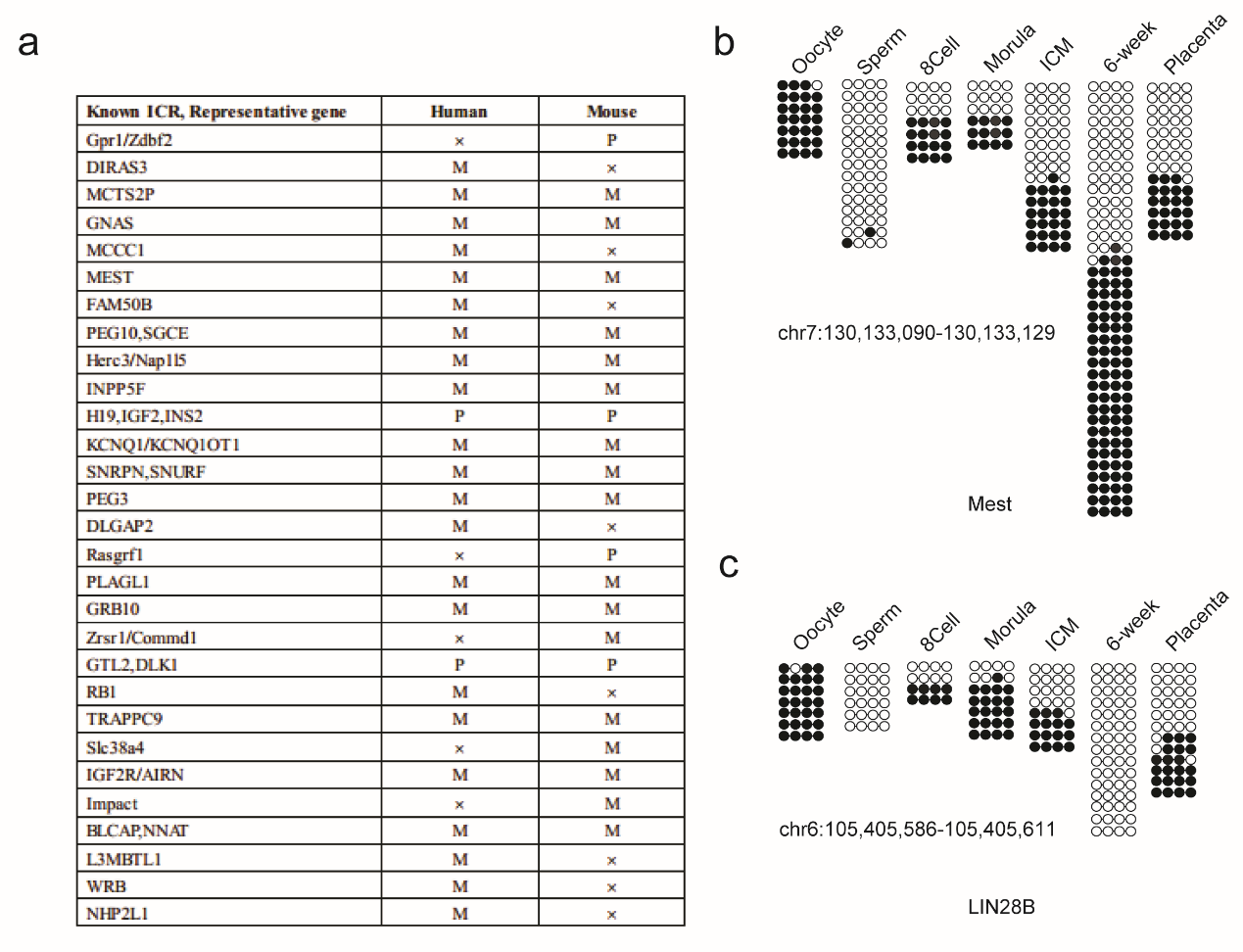
**

**Figure S5** Genomic imprinting in mammals. **(a)** Well-validated germline ICRs in humans and mice. “M” means maternally imprinted, “P” means paternally imprinted, and “×” means not imprinted. **(b-c)** Representative changes in DNA methylation for two loci located in the germ-line ICR of *MEST* (b) and the placenta-specific ICR of *LIN28B* (c). Paired reads were extracted and merged to track the dynamics of each site. Open circles represent unmethylated CpGs, and filled circles represent methylated CpGs.
