# Supplementary material for: DNA methylation reprogramming of functional elements during mammalian embryonic development
Source: Cell Discov. 2018 Aug 7;4:41. doi: 10.1038/s41421-018-0039-9 (PMC6079081; doi:10.1038/s41421-018-0039-9)
Supplement: Supplementary file 2 — Table S1 [file 41421_2018_39_MOESM2_ESM.pdf]

Supplemental Tables

Table S1. Summary of shotgun bisulfite sequencing

Stage	Replication	BSCR (%)	# CpG	Raw Depth	Dedup Depth	1× Cov (%)	3× Cov (%)	5× Cov (%)
**Oocyte**	Rep1	98.9	26,157,159	71.0	6.1	92.7	67.9	49.5
	Rep2	99.0						
**Sperm**	Rep1	99.1	27,416,714	34.2	33.0	97.2	96.7	95.9
	Rep2	99.3						
	Rep3	99.3						
**2-cell**		98.6	14,885,465		2.73	50.6	19.0	7.9
**8-cell**	Rep1	99.2	26,911,155	15.2	6.3	94.6	77.3	51.0
	Rep2	99.0						
**Morula**	Rep1	98.8	27,528,048	27.7	10.1	97.6	94.1	86.1
	Rep2	99.3						
**ICM**	Rep1	99.1	27,474,837	25.4	10.2	97.4	93.4	85.7
	Rep2	98.8						
**6-week**	Rep1	99.1	27,596,867	32.1	31.7	97.8	97.2	96.6
	Rep2	99.1						
	Rep3	99.1						
**Placenta**	Rep1	99.1	27,499,530	21.1	19.5	97.5	96.4	94.9
	Rep2	98.8						

"BSCR" means the bisulfite conversion rate calculated by spike-in unmethylated lambda DNA. "Raw Depth" was calculated from the uniquely mapped reads before removal of PCR duplication. "Dedup Depth" indicates the sequencing depth of CpGs for two strands from the uniquely mapped reads after removal of PCR duplication. "Cov" means the proportion of mapped CpGs over total CpGs in human genome.

Table S2. GO enrichment analyses for human promoters.

Table S3. Methylation levels of enhancers for both human and mouse during early embryogenesis.

Table S4. GO enrichment analyses for human enhancers.

Table S5. GO enrichment analyses for mouse enhancers.

Table S6. Methylation states of imprinting control regions in gametes, early embryos, placenta and skin.
