# Supplementary material for: DNA methylation reprogramming of functional elements during mammalian embryonic development
Source: Cell Discov. 2018 Aug 7;4:41. doi: 10.1038/s41421-018-0039-9 (PMC6079081; doi:10.1038/s41421-018-0039-9)
Supplement: Supplementary file 3 — Table S2 [file 41421_2018_39_MOESM3_ESM.pdf]

Table S2. GO enrichment analyses for human promoters.

Category	Term
GOTERM_BP_FAT	GO:0007601~visual perception
GOTERM_BP_FAT	GO:0050953~sensory perception of light stimulus
GOTERM_BP_FAT	GO:0006096~glycolysis

Count	%	PValue	Genes	List Total	Pop Hits	Pop Total	Fold Enrich
3	4.347826	0.082468039	810815, 78	31	216	13528	6.060932
3	4.347826	0.082468039	810815, 78	31	216	13528	6.060932
2	2.898551	0.099245035	786243, 81	31	47	13528	18.56966

Bonferroni	Benjamini	FDR
1	1	68.76456
1	1	68.76456
1	1	75.66154
